# The pre-existing population of 5S rRNA effects p53 stabilization during ribosome biogenesis inhibition

**DOI:** 10.18632/oncotarget.13833

**Published:** 2016-12-09

**Authors:** Carmine Onofrillo, Alice Galbiati, Lorenzo Montanaro, Massimo Derenzini

**Affiliations:** ^1^ Department of Experimental, Diagnostic and Specialty Medicine (DIMES), University of Bologna, S. Orsola Hospital, 40138, Bologna, Italy

**Keywords:** ribosome biogenesis inhibition, 5S rRNA, ribosomal proteins, MDM2, p53

## Abstract

Pre-ribosomal complex RPL5/RPL11/5S rRNA (5S RNP) is considered the central MDM2 inhibitory complex that control p53 stabilization during ribosome biogenesis inhibition. Despite its role is well defined, the dynamic of 5S RNP assembly still requires further characterization. In the present work, we report that MDM2 inhibition is dependent by a pre-existing population of 5S rRNA.

## INTRODUCTION

Proliferating cells are characterized by elevated production of cellular components needed for the survival of new born cells. Protein synthesis is necessary not only to ensure cellular functions but also to achieve the proper outcome for cell division. Therefore, regulation of ribosome biogenesis and cell cycle must be tuned to guarantee the survival of doubling cells [1]. Different studies described how positive stimuli like nutrient, growth factors, cytokines and mitogens increase both ribosome biogenesis and protein synthesis to allow appropriate cell growth and proliferation. In contrast, under stress situation like starvation, hypoxia or DNA damage, proliferating cells negatively modulate ribosomes production to reduce protein synthesis and block cell cycle progression [2]. The main strategy used by cycling cells to coordinate cell proliferation and ribosome biogenesis is to share regulatory elements [3]. In eukaryotes, ribosome biogenesis requires the activity of all the RNA Polymerases. In particular, RNA polymerase I (POLI) regulates the transcription of a precursor rRNA (45S), which after a multi-step processing, give rise to the maturation of three rRNA species 28S, 5.8S and 18S. The transcription of ribosomal proteins mRNAs is dependent by RNA polymerase II (POLII), while RNA polymerase III (POLIII) is responsible for 5S rRNA, tRNAs and some small nuclear RNAs transcription [4, 5]. While 28S, 5.8S, 5S rRNA and large ribosomal proteins constitute (LRPs) the 60S subunit, the 40S subunit is composed by 18S rRNA and small ribosomal proteins (SRPs). Several oncogenes, like c-Myc, are able to enhance the production of ribosomal components, by the stimulation of all the three RNA Polymerases [6–9], while tumor suppressors like p14 ARF or p53, are able to bind and inhibit the activity of several transcription factors like SL1 and UBF, needed for Polymerase I transcription, leading to ribosome biogenesis inhibition [10, 11]. On the other hand, several alterations in ribosome biogenesis, such as rRNA transcription inhibition or rRNA processing impairment, lead to a coordinated induction of p53 activity. In fact, different ribosomal proteins such as RPL11, RPL4, RPL5, RPL23, RPL37, RPS15, RPS20,RPS27a, RP27,RPS27l, RPS25 exert extraribosomal functions and are able to bind and inhibit the E3 ubiquitinase Mouse Double Minute 2 (MDM2) leading to the stabilization of the tumor suppressor p53 [12–21]. Recent studies better characterized the ribosomal proteins/MDM2/p53 axis and identified the 5S rRNA a mandatory factor for p53 stabilization in response to ribosome biogenesis inhibition.

In particular, it has been demonstrated that 5S rRNA is present in a 60S preribosomal complex with RPL5 and RPL11, defined as 5S RNP, which is presently considered as the key regulator of MDM2 inhibition [22, 23, 24]. There is evidence that 5S rRNA and RPL5 are very abundant in the nuclear fraction respect to RPL11, which accumulates in the nucleoplasm only when ribosome biogenesis is blocked [22]. This may suggest that MDM2 inhibition could be dependent by 5S RNP assembly during ribosome biogenesis inhibition. In the present work we found that 5S rRNA neosynthesis inhibition do not efficiently affect p53 stabilization after inhibition of rRNA transcription. Thus, in order to verify if MDM2 inhibition can be dependent by a pre-existing rather than a newly synthesized 5S rRNA fraction, we measured both the populations bound to MDM2 during rRNA transcription inhibition. We found that pre-existing 5S rRNA represents the major fraction bound to MDM2 under ribosomal stress, thus indicating its importance in p53 stabilization. Moreover, our results suggested that RPL5 and RPL11 are both necessary for 5S rRNA accumulation in cells. Indeed, the recruitment of the pre-existing population of 5S rRNA, allows the efficient inhibition of the increasing MDM2 molecules, during ribosome biogenesis inhibition.

## RESULTS

### TFIIIA depletion leads to 5S rRNA neosynthesis inhibition

Previous works have been characterized the involvement of 5S RNP in p53 stabilization after ribosome biogenesis inhibition. In particular, it has been demonstrated that RPL11, RPL5 and 5S rRNA act as a ternary complex in a mutually dependent manner [23]. On the other hand, 5S rRNA and RPL5 are very abundant in non-ribosomal fraction and distributed between nucleolus, nucleoplasm and cytoplasm, while RPL11 is principally present in the nucleolus and cytoplasm, without any accumulation in the non-ribosomal fraction. Moreover, after ACTD treatment, the level of non-ribosomal RPL11 considerably increased in the nucleoplasm, in contrast to 5S rRNA and RPL5 [22]. To better clarify the dynamics of 5S RNP assembly, in the present work we investigated the recruitment of neo-synthesized and pre-existing 5S rRNA populations to MDM2 binding during ribosome biogenesis inhibition.

**Figure 1:**
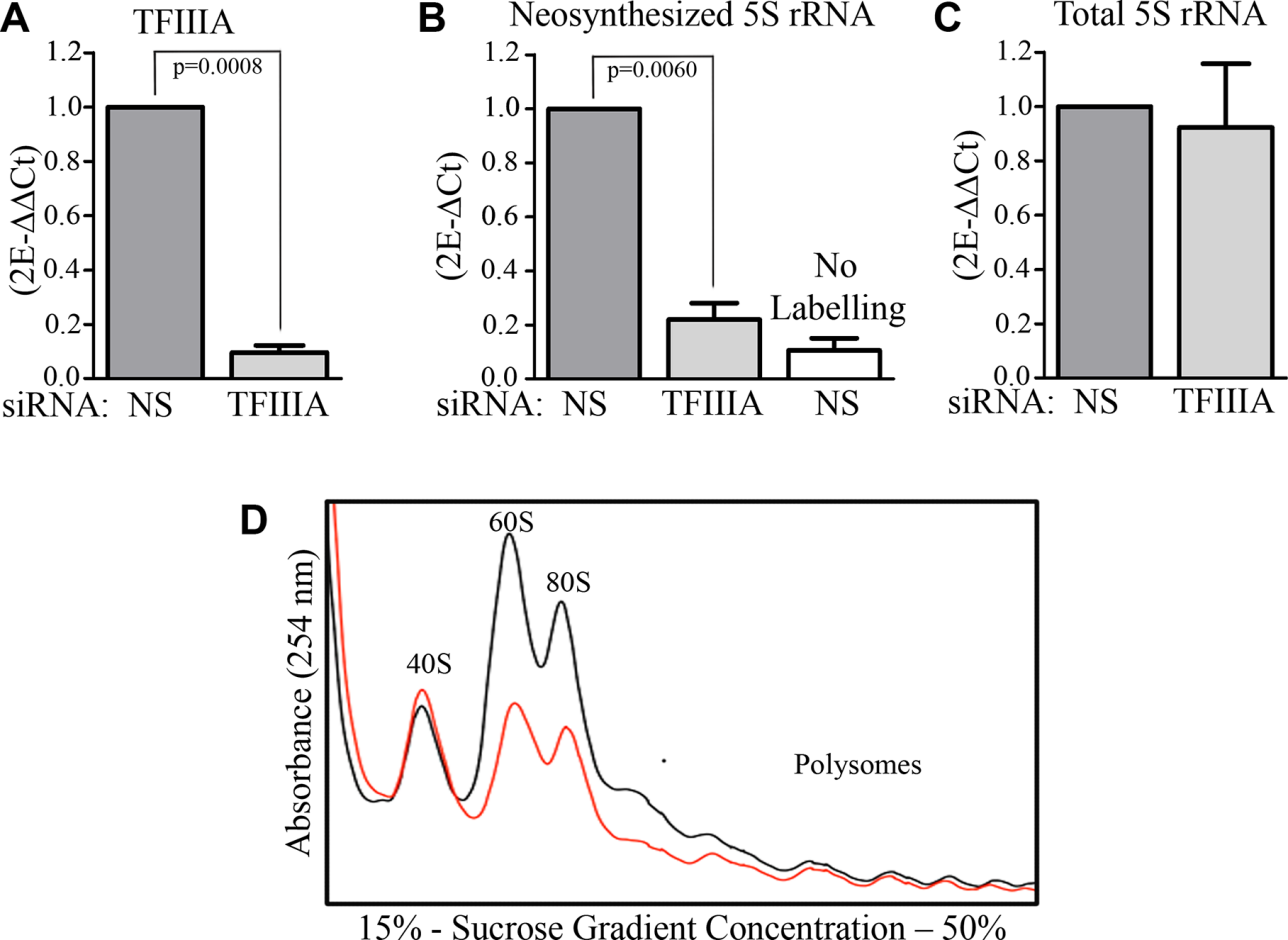
TFIIIA depletion lead to 5S rRNA neosynthesis inhibition (**A**) Real time qPCR of TFIIIA mRNA 72 hours after the beginning of transfection procedure with specific siRNA against TFIIIA mRNA (siTFIIIA) or non silencing siRNA in HCT116 cells. Graph bars represent the mean ± SEM of three independent experiments. (**B**) Real time qPCR of captured 5-EU labeled 5S rRNA. 72 hours after the beginning of TFIIIA interference, HCT116 cells were labeled with 5-EU for 1 hour and RNA was extracted from siNS, siTFIIIA and from an unlabeled sample, after a chase time of 2 hours with Uridine 1 mM, as indicated above by the representation of the experimental design. All the extracted RNA were modified with biotin azide, captured by streptavidin magnetic beads, reverse-trascribed into cDNA and 5S rRNA relative sequence was amplified by qPCR. Graph bars represents mean ± SEM of three independent experiments. (**C**) Real time qPCR of total 5S rRNA 72 hours after the beginning of transfection procedure with siTFIIIA or siNS, in HCT116 cells. Graph bars represent the mean ± SEM of three independent experiments. (**D**) Polysome profile analysis of whole HCT116 extracts separated by sucrose gradient centrifugation. Samples were collected 72 hours after siNS or siTFIIIA transfection.

In a preliminary set of experiments, we set up the conditions to down-regulate the expression of 5S rRNA, according to previous studies [22, 23]. In order to inhibit the transcription of 5S rRNA by RNA Polymerase III (POLIII), we employed a siRNA against TFIIIA, the transcription factor responsible for POLIII specific recruitment on 5S rRNA promoter, as described previously. After 72 hours from the beginning of the transfection procedure, we measured the level of TFIIIA expression in human derived Colon Carcinoma cells (HCT116), by RT-qPCR, obtaining an efficient inhibition of about 90% respect to control cells (Figure 1A), transfected with non-silencing siRNA (NS). After TFIIIA interference, the amount of newly synthesized 5S rRNA was efficiently reduced, as shown by the analysis of captured 5-Ethynyl Uridine (5-EU) labeled 5S rRNA via click chemistry based approach (RNA nascent capture kit, Thermo scientific). 5S rRNA neosynthesis inhibition was also confirmed by autoradiographic analysis (Supplementary Figure S1A). In contrast, total 5S rRNA amount is not significatively affected by TFIIIA interference (Figure 1C), despite the 60S production reduction observed by polysome profile analysis (Figure 1D) and the failure of 28S processing by auto radiographic analysis of rRNA precursors (Supplementary Figure S1B), Thus suggesting that a 5S rRNA non-ribosomal fraction is still present in cells after TFIIIA inhibition.

### 5S rRNA neosynthesis inhibition do not efficiently abrogates p53 stabilization after inhibition of ribosome biogenesis

**Figure 2:**
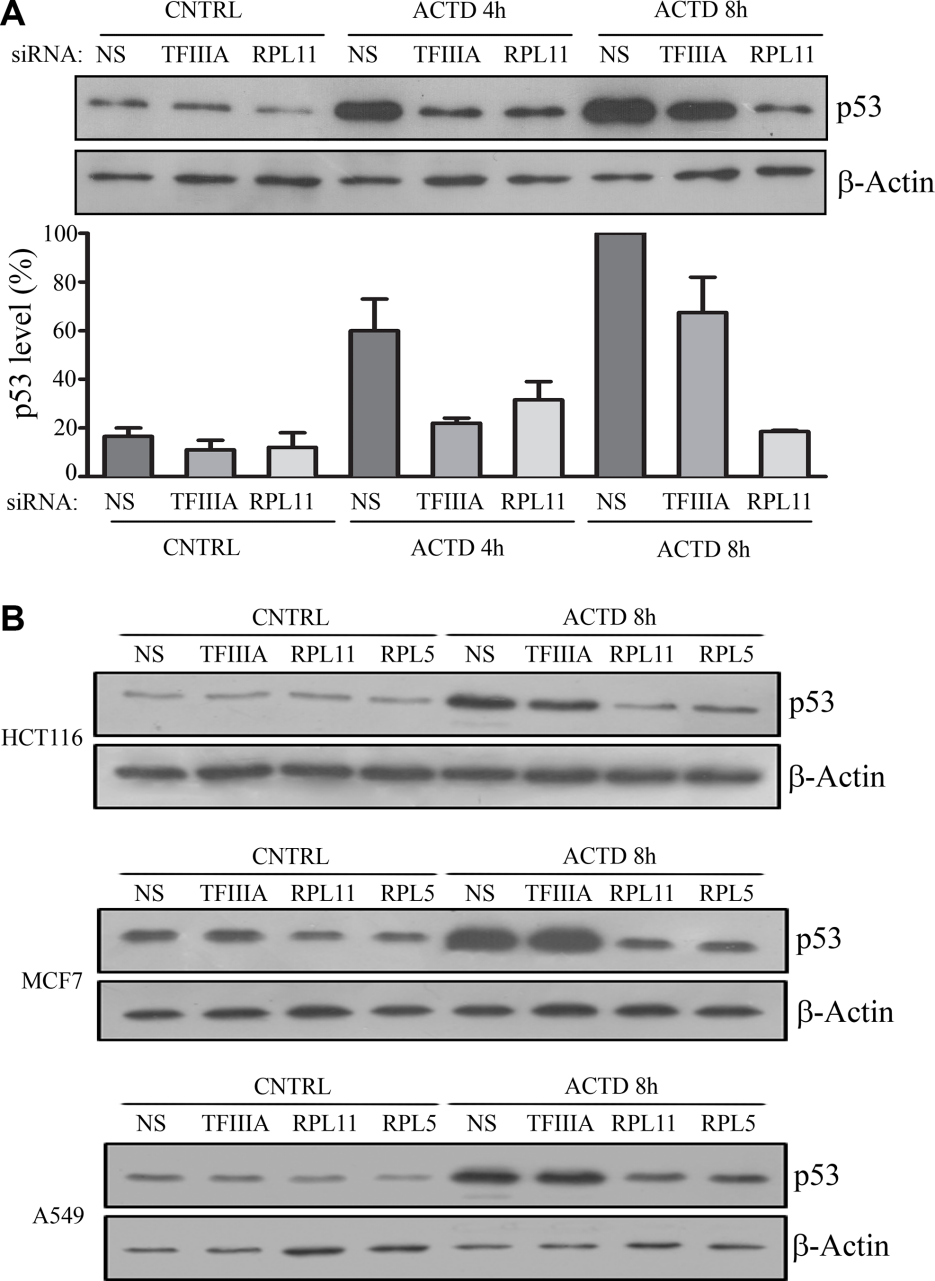
5S rRNA neosynthesis inhibition do not efficiently abrogates p53 stabilization after inhibition of ribosome biogenesis (**A**) Western blot analysis of p53, and β-actin as loading control, in HCT116 cell. Cells were treated with ACTD for 4 or 8 hours at 8 nM, 72 hours after the beginning of siNS, siTFIIIA and siRPL11 transfection procedure, as indicated in the upper panel. Lower panel represents the densitometric analysis of p53/β-actin ratio in which the indicated value are normalized for the maximum level of measured p53 (NS, ACTD 8 h). Graph bars represents mean ± SEM of three independent experiments. (**B**) Western blot analysis of p53, and β-actin as loading control, in HCT116, MCF7 and A549 cells, which were treated with ACTD for 8 hours at 8 nM, 72 hours after the beginning of siNS siTFIIIA, siRPL11 or RPL5 transfection procedure, as indicated.

In order to verify how 5S rRNA neosynthesis inhibition affects p53 stabilization, we treated TFIIIA-silenced HCT116 cells with Actinomycin D (ACTD, 8 nM) for 4 and 8 hours. Western blot analysis indicated that p53 levels are in general much lower in TFIIIA silenced samples, in comparison with control cells. However, by treating cells with ACTD, the amount of p53 progressively increased, despite the inhibition of 5S rRNA transcription (Figure 2A). In contrast, this was not the case in cells in which the expression of RPL11 or RPL5 was reduced by RNA interference.

In fact, in HCT116 cells, RPL11 RNA interference (Figure 2A, Supplementary Figure S2) abrogated the progressive stabilization of p53, as observed also after RPL5 interference in HCT116, MCF7 and A549 cells (Figure 2B, Supplementary Figure S2).

This data was confirmed by using a different set of siRNA against TFIIIA and RPL11 mRNAs (Supplementary Figure S3).

Moreover, a slighter difference between TFIIIA and RPL11 or RPL5 depleted samples was observed in terms of p53 stabilization after the selective inhibition of POLI transcription by treatment with the non-intercalating agent CX-5461 (Supplementary Figure S4).

These data suggest that the suppression of 5S rRNA synthesis do not abrogate p53 response to inhibition of ribosome biogenesis, in contrast to RPL11 and RPL5 reduction.

### After ribosome biogenesis inhibition, the assembly of the 5S RNP complex occurs under a reduction of 5S rRNA neosynthesis

The above reported data indicated that during ACTD treatment, p53 stabilization occurred also in absence of 5S rRNA neosynthesis. In order to ascertain whether in this case the 5S rRNA no longer constitute the 5S RNP complex, we performed TFIIIA RNA interference in HCT116 cells and we evaluated the amount of 5S rRNA co-immunoprecipitated with MDM2 at 4 and 8 hours of ACTD treatment.

**Figure 3:**
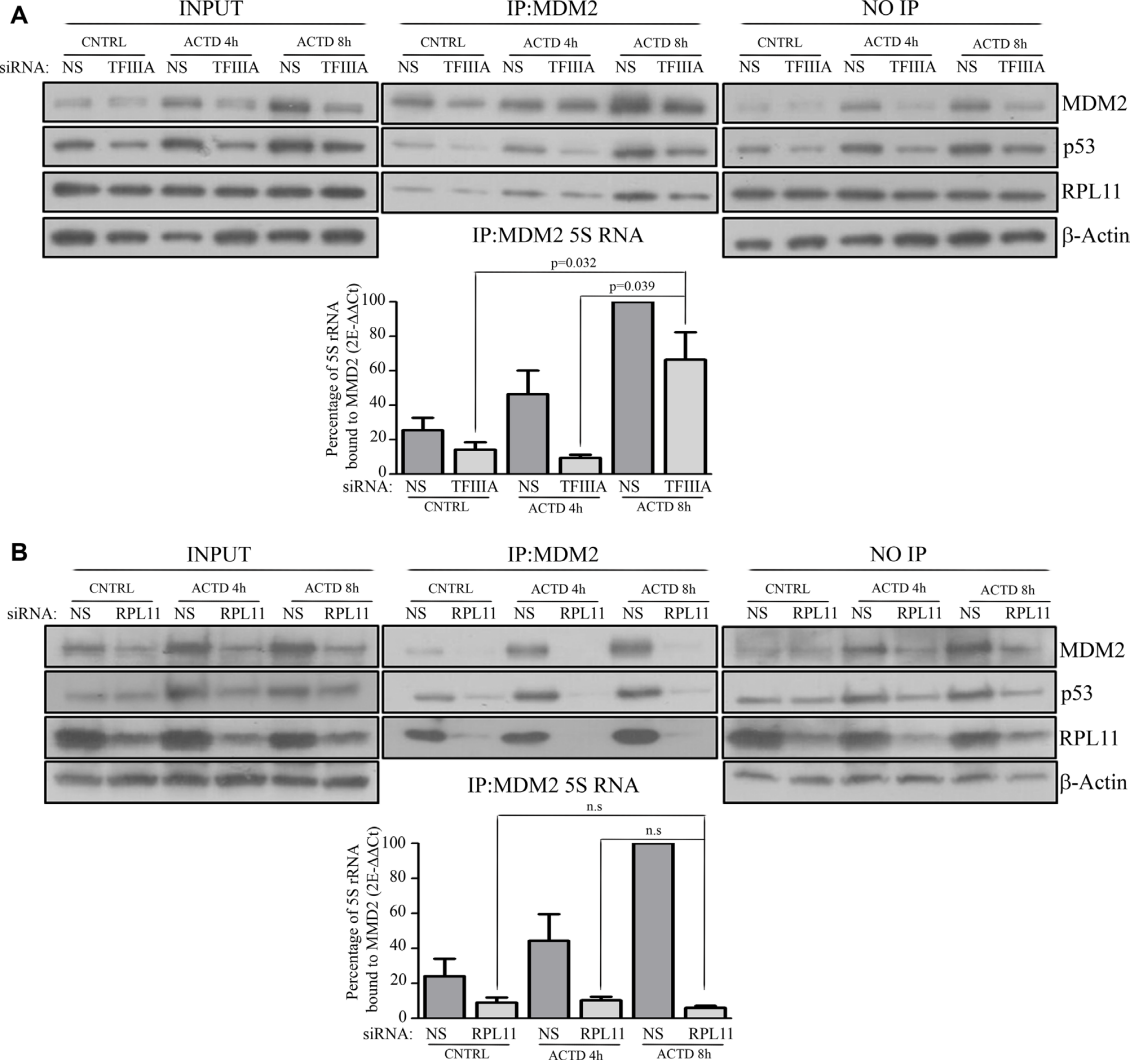
After ribosome biogenesis inhibition the assembly of the 5S RNP complex occurs under a reduction of 5S rRNA neosynthesis (**A** and **B**) upper panels: Western blot analysis of HCT116 MDM2-immunoprecipitated whole extract after siNS and siTFIIIA (A, upper panel) or siRPL11 (B, upper panel), treated with ACTD (8nM) for 4 or 8 hour 72 hours after the beginning of the transfection procedure, as indicated. Representative Western blots show the level of MDM2, p53, RPL11 and β-actin from whole extract (INPUT) and non immunoprecipitated proteins derived from the immunoprecipitation supernatant (NO-IP), while MDM2, p53 and RPL11 amount are shown for MDM2 immunoprecipitated samples (IP). (**A** and **B**) lower panels: RT-qPCR of MDM2 co-immunoprecipitated 5S rRNA. HCT116 cells were treated with ACTD (8nM) for 4 or 8 hours 72 hours after the beginning of siNS and siTFIIIA (A, lower panel) or siRPL11( B, lower panel) transfection procedure. The indicated value are normalized for the maximum level of immunoprecipitated MDM2 (NS, ACTD 8 h). Graph bars represents mean ± SEM of three independent experiments.

The data obtained showed that, in control cells, ACTD treatment led to a progressive time dependent increase of co-immunoprecipitated 5S rRNA and MDM2 (Figure 3A, Figure 3B). Interestingly, also in TFIIIA silenced cells, we found a similar correlation, thus indicating that at 8 hours of ACTD treatment the 5S rRNA bound to MDM2 was not the neo-synthesized fraction. Moreover, RPL11 was co-immunoprecipitated with MDM2 and 5S rRNA despite TFIIIA silencing, thus suggesting that the formation of the typical 5S RNP complex occurred (Figure 3A). In contrast, after reducing RPL11 expression by RNA interference, we observed a clear reduction of 5S rRNA and MDM2 co-immunoprecipitated after ACTD treatment for both 4 or 8 hours, indicating that the RPL11 newly synthesized fraction is involved in p53 stabilization (Figure 3B). Taken together, these data indicated that the reduction of 5S rRNA neosynthesis does not efficiently hinder 5S RNP binding to MDM2 after ribosome biogenesis inhibition.

### Pre-existing 5S rRNA is recruited for MDM2 binding during ribosome biogenesis inhibition

The data reported above suggest that a non-ribosomal fraction of 5S rRNA, stored in the cell, take part in the assembly of the 5S RNP complex during a prolonged inhibition of rRNA transcription. To verify this hypothesis, we measured changes in different populations of 5S rRNA bound to MDM2 under ribosome biogenesis inhibition.

**Figure 4:**
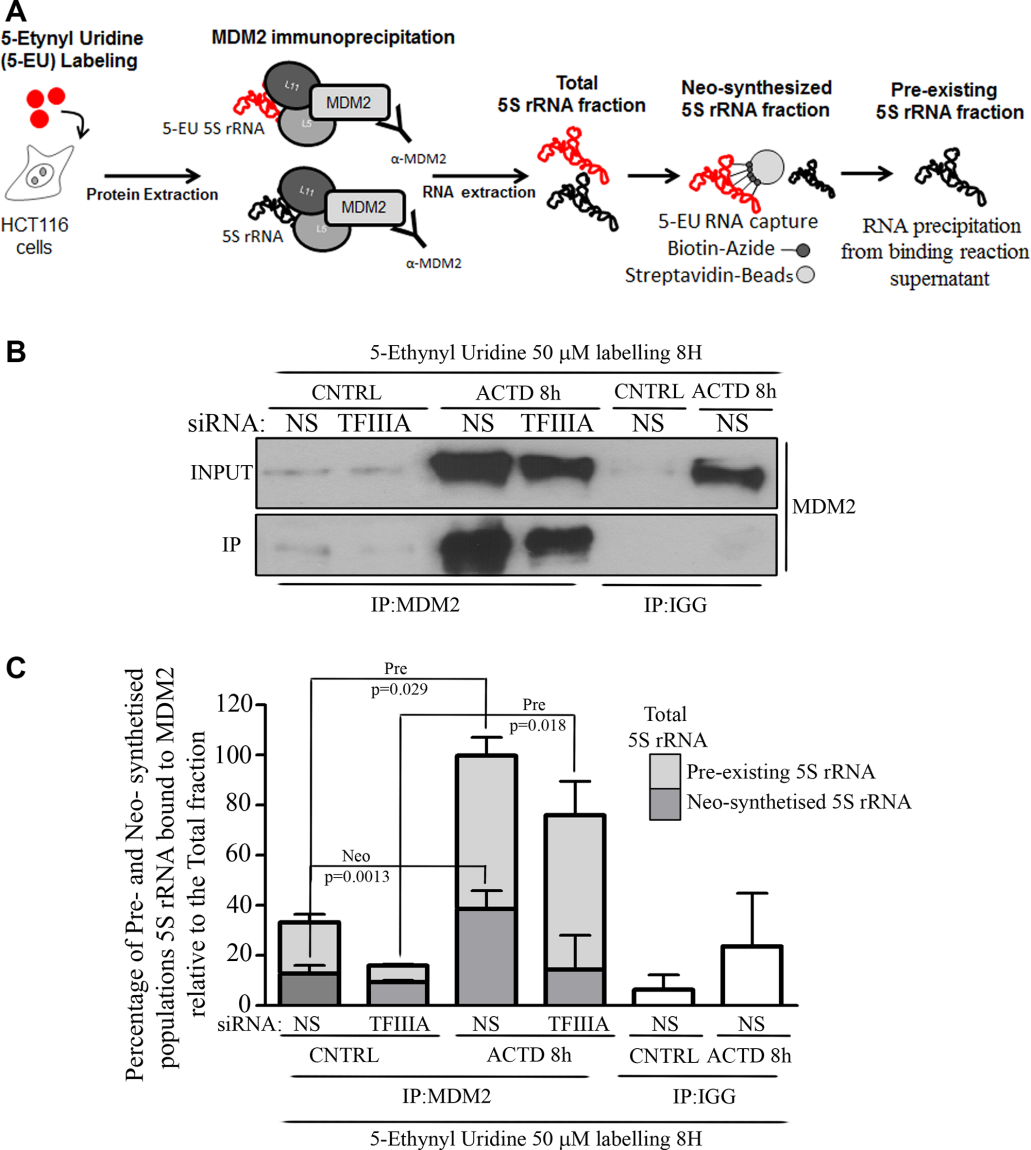
Pre-existing 5S rRNA is recruited to MDM2 binding during ribosome biogenesis inhibition (**A**) Graphical experimental design relative to the collection of 5S rRNA fractions from MDM2 immunoprecipitated samples. We labeled HCT116 cells with 5-EU and we performed MDM2 immunoprecipitation on the whole protein extract. The RNA extracted from immunoprecipitated samples was in turn subjected to the capturing of labeled fraction (by Click-it Nascent RNA capture kit, Life technologies, Eugene Oregon, USA) with the collection of the unlabeled fraction or was used directly as a total RNA fraction. RNA fractions were reverse-transcribed and qPCR was performed in order to measure the different amount of 5S rRNA (see also materials and methods). (**B**) Western Blot analysis of MDM2 immuoprecipitation. Whole protein extract was collected from HCT116 cells treated with ACTD (8nM) for 8 hours, 72 hours after siNS or siTFIIIA transfection procedure. Representative Western blots show the level of MDM2 both in INPUT or MDM2 and IGG immunoprecipitated samples (IP). (**C**) Fold changes of neo-synthesized and pre-existing 5S rRNA fractions relative to total 5S rRNA variations bound to MDM2 after TFIIIA interference and ACTD treatment. HCT116 cells were labeled for 8 hours, as indicated above by the experimental design. Whole protein extraction was immunoprecipitated with an antibody against MDM2 and RNA was extracted. Labeled and unlabeled RNA fraction was isolated via click chemistry approach and neo-synthesized, pre-existing and total 5S rRNA amount was evaluated by RT-qPCR (see also experimetal procedures). Graph bars represent mean ± SEM of three independent experiments.

In particular, we labeled HCT116 cells with 5-Ethynyl Uridine (5-EU) in a dose which did not stabilize p53 (Supplementary Figure S5) for 8 hours and in presence or absence of ACTD treatment. As represented in Figure 4A, we extracted RNA from MDM2 immunoprecipitated samples (total fraction), then we captured the labeled fraction (neo-synthesized fraction) via click chemistry approach (Nascent RNA capture kit^®^, Life technologies, Eugene, Oregon, USA) and collected the unlabeled fraction from the supernatant of the capturing phase (pre-existing fraction).

Following this approach we observed that after ACTD treatment, the increase in the total amount of MDM2 immunoprecipitated 5S rRNA is mainly dependent by the pre-existing population of 5S rRNA rather than the neo-synthesized captured fraction, even under 5S rRNA neosynthesis inhibition (Figure 4B and 4C).

These results demonstrate that under ribosome biogenesis inhibition, in addition to neo-synthesized 5S rRNA, a pre-existing fraction of 5S rRNA is recruited in 5S RNP, ensuring the efficient inhibition of all the increasing MDM2 molecules.

## DISCUSSION

Here we reported that 5S rRNA synthesis did not abrogate p53 accumulation due to ActD treatment, in contrast to RPL5 or RPL11 neosynthesis inhibition.

Therefore, this data might suggest that the stabilization of p53 after inhibition of ribosome biogenesis can occur without the contribution of 5S rRNA, being exclusively due to RPL11 and RPL5 MDM2 binding.

This appears to be in contrast with the observation that under a prolonged down-regulation of rRNA synthesis by POLR1A RNA interference, p53 stabilization was induced as a consequence of MDM2 inhibition by the 5S RNP ternary complex which contains RPL11, RPL5 and 5S rRNA [23].

In order to clarify this apparent discrepancy, we measured the total amount of 5S rRNA bound to MDM2 during a progressive hindering of ribosome biogenesis by ActD treatment. We found that, despite the inhibition of 5S rRNA synthesis, a progressive increase of 5S rRNA co-immunoprecipitated with MDM2 occurred. These results confirmed that during ribosome biogenesis inhibition the 5S rRNA is necessary for the formation of the 5S RNP, thus suggesting that a pre-existing population of 5SrRNA is involved in 5S RNP assembly.

To verify this point, we measured the amount of total, neo-synthesized and pre-existing 5S rRNA from MDM2 immunoprecipitated samples after ActD treatment.

The data obtained showed that under inhibition of ribosome biogenesis, the increase in total 5S rRNA co-immunoprecipitated with MDM2 observed is mainly dependent by the pre-existing population, even after TFIIIA interference. Since RPL5 and RPL11 silencing led to a strong inhibition of p53 induction after ACTD treatment, it was very likely that the newly synthesized RPL5 and RPL11 are responsible for stored 5S rRNA recruitment and accumulation in cells. On the other hand, data indicated that RPL5 and RPL11 exert different dynamics relative to 5S RNP assembly. In fact, It has been shown that RPL11 is localized principally in the nucleolus and cytoplasm and significantly increases in nucleoplasm only after ACTD treatment, in contrast to RPL5 and 5S rRNA which are accumulated between nucleolus, nucleoplasm and cytoplasm.

Moreover, RPL5 is required in 5S rRNA maturation [22] and accumulates with 5S rRNA in a non-ribosomal pre-5S RNP complex [24, 25], while the accumulation of RPL11 in the nucleus was related to PICT1 activity [26, 27], which was identified as a mediator of 5S RNP incorporation into nascent 60S subunit [25].

**Figure 5:**
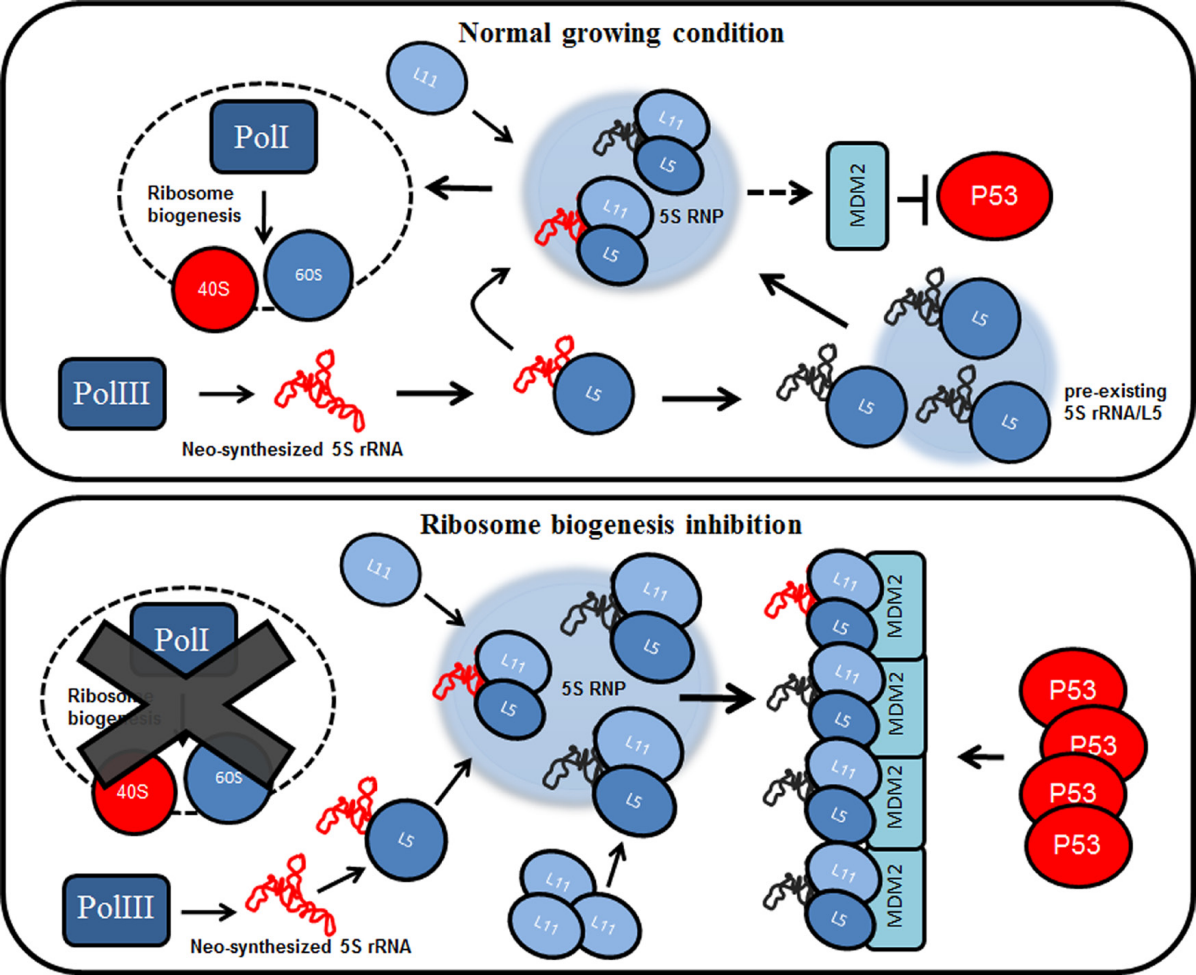
The pre-existing population of 5S rRNA effects p53 stabilization during ribosome biogenesis inhibition

In order to better characterize the role of RPL11 and RPL5 in 5S RNP assembly during a progressive inhibition of ribosome biogenesis, we compared the amount of RPL11 and RPL5 in cytoplasmic and nuclear extracts from HCT116 cells after ACTD treatment. Our data demonstrated that the amount of RPL11 in the nucleus was increased after 8 hours of ACTD administration. In contrast, we found that the amount of RPL5 in the nuclear extract remained constant during ACTD treatment (Supplementary Figure S6). While this data are in contrast to previous studies [28], they reflect other experimental observations [29, 21]. The observed stability of RPL5 distribution in the nuclear fraction could reflect its role in premature binding of 5S rRNA and in the accumulation of the pre-5S RNP complex. Since the inhibition of RPL5 expression abrogated the progressive stabilization of p53 under ribosome biogenesis inhibition, it is very likely that the newly synthesized RPL5 continuously bind 5S rRNA, thus allowing a progressive storage of a pre-5S RNP complex. However, we found that 5S rRNA stability is dependent by both RPL5 and RPL11 synthesis, since after their inhibition by RNAi, the amount of neo-synthesized 5S rRNA is drastically reduced (Supplementary Figure S7). Thus, the absence of RPL11 or RPL5 synthesis would abrogate the assembly of pre-5S RNP complex, possibly due to their mutual protection [28]. This may well explain why the inhibition of RPL5 or RPL11 expression, but not that of 5S rRNA, hinders p53 stabilization also after long-term rRNA synthesis inhibition. The observation that RPL11 progressively accumulates in the nucleus during the inhibition of rRNA transcription, suggest that after ribosome biogenesis blockage the availability of newly synthesized RPL11 could control the formation of the 5S RNP complex by recruiting the stored pre-5S RNP complex (for a schematic representation see Figure 5). Our results shed new light on the dynamics of 5S RNP complex assembly under inhibition of rRNA transcription.

## MATERIALS AND METHODS

### Cell lines, culture conditions and siRNA transfection

Human cancer-derived cell lines were cultured in monolayer at 37°C in humidified atmosphere containing 5% CO2. HCT116, and A549 were cultured in Dulbecco's modified Eagle's medium (Sigma Aldricth, Saint Louis, MO, USA), while MCF7 in RPMI medium (Sigma Aldricth, Saint Louis, MO, USA) supplemented with 10% fetal bovine serum (FBS, Sigma Aldrich, Saint Louis, MO, USA). Actinomycin D (Sigma Aldrich, Saint Louis, MO, USA) was used at a final concentration of 8 nM for the indicated times. siRNA transfection was performed by Lipofectamine RNAi Max and OPTIMEM medium (Applied Biosystems, Foster City, CA, USA) as manufacturer's protocol. Each siRNA was transfected at a concentration of 50 nM and cells are always treated for the indicated times after 72 h from the beginning of the transfection procedure. Two set of siRNAs were used. The sequences of the first set, targeting TFIIIA, RPL11 and RPL5 mRNA are listed in supplemental materials and methods section, while non silencing siRNA (Quiagen, Hilden, Germany), was used as transfection control. For the second set, non silencing siRNA, or sequences targeting TFIIIA or RPL11 were purchased from Life technologies, Eugene, Oregon, USA.

### RNA extraction, reverse transcription and real time qPCR

RNA extraction from whole cell lysate or from immunoprecipitated samples was performed using Tri Reagent (Ambion, Austin, TX, USA) according to the manufacturer's protocol. Reverse transcription was performed using High Capacity cDNA Reverse Transcription Kit (Applied Biosystems, Foster City, CA, USA) following the manufacturer's protocol. The relative amounts of TFIIIA mRNA, RPL11 mRNA, RPL5 mRNA, 5s rRNA was evaluated by Sybr Green method (Applied Biosystems, Foster City, CA, USA) using primer's sequences listed in supplemental materials and methods section, while b-glucoronidase was used as standard control and quantified with TaqMan Gene expression assay (Applied Biosystems, Foster City, CA, USA). Real time qPCR was performed on an ABI prism 7000 Sequence Detection system (Applied Biosystems, Foster City, CA, USA) with the 2E−ΔΔCT method. The mean ΔCT value of the control sample was used in each experiment to calculate the ΔΔCT value of sample replicates. Statistical analysis on the data obtained was performed by using the paired *T*-Test on three experimental replicates by considering significative data with a *P value* < 0,05.

### Autoradiographic analysis of ^3^H-Uridine labeled rRNA

For 18S and 28S rRNA processing analysis or evaluation of neo-synthesized 5S rRNA and tRNAs, pulse-chase labeling was performed. In brief, 72 hours after transfection with siRNA against TFIIIA or NS siRNA, HCT116 cells were incubated in medium containing 2,5 μCi/mL of ^3^H-Uridine for 1 hour (indicated as Pulse). Cells were then changed to medium containing non-radioactive Uridine at the concentration of 1 mM and harvested after 4 h (here described as Chase). Total RNA was isolated with TRIreagent (Ambion, Austin, TX, USA) and the extracted RNA was quantified by spectrophotometric analysis and the radioactivity incorporation was evaluated with β-Counter analysis. For 18S and 28S rRNA processing analysis, we collected samples both after 1h of Pulse labeling or after a chase time of 4 h and equal amount of ^3^H-Uridine labeled RNAs were resolved in Formammide loading Dye (25% Formammide; 3,3 mM EDTA PH 8; 85 ug/ml Blue Bromophenolo/Xylene Cyanol) by 1% agarose–formaldehyde gel (in 1X MOPS/6% Formaldehyde running buffer, with EtBr at 0,05 mg/ml ). For small RNA analysis, samples were collected after a chase time of 4h and electrophoresed in Formammide Loading Dye on a 10% polyacrylamide (19:1)/TBE/7M Urea Gel in TBE 0.5X running buffer at 200V constant for 1h. At the end of the run, the gel was stained with etBr at 0.5 mg/ml in TBE 0.5X for 10 min at room temperature while shaking and destained for 10 min in TBE 0.5X at room temperature while shaking. For both RNA electrophoresis, relative images were collected on UV transillumiator. Labeled RNA was then transferred on Hybond N+ membrane (GE Healthcare, Little Chalfont, Buckinghamshire, UK) by semidry transfer in TBE 0.5X for small rRNAs or by backward capillarity transfer in 20X SSC for 18S and 28S evaluation. RNAs were UV-crosslinked to the membrane by Stratalinker^®^ UV Crosslinker and the membranes was treated with EN^3^HANCE™ Spray Surface Autoradiography Enhancer (Perkin Elmer, Waltham, MA, USA). Finally, for labeled RNAs autoradiographic detection, the membranes were exposed for 6 days at −80°C on ECL Amersham Hyperfilms (GE Health Care, Little Chalfont, Buckinghamshire, UK).

### Polysome profile analysis

HCT116 cells were cultured in 150 mm dishes and treated with 100 μg/ml Cycloheximide for 15 min, 72 hours after the beginning of the transfection procedure. Cells were then washed twice on ice in PBS + 100 μg/ml Cycloheximide (CHX); resuspendend in LSB buffer (20 mM TRIS HCL, PH 7.5; 10 mM NaCl; 3 mM MgCl2; 100 μg/ml CHX; 0,04 U/μl RiboLock RNAsi inhibitor [Thermo Scientific, Waltham, MA, USA]; Protease inhibitor cocktail [Roche Diagnostics, Basel, Switzerland]) and lysate on ice for 10 min. by adding detergent buffer (0,3% Triton N101 50 mM Sucrose; 100 μg/ml CHX; 0,04 U/μl RiboLock RNAsi inhibitor (Thermo Scientific, Waltham, MA, USA) The lysate was cleared by centrifugation at 14000 RCF for 10 min. at 4°C. Ribosomes was then separated on chilled 15%–50% Sucrose Gradient (in LSB buffer) by ultracentrifugation at 160000 G for 2 hours at 4°C. Polysome Profile was monitored at 254 nm (0,2 OD sensibility) and fractionated (at 10X, 10% TRIS-Pump power) using an ISCO gradient fractionator system interfaced to an UA-6 absorbance detector (Teledyne Isco, Lincon, NE, USA). Collected data were digitally converted by using Minilab 1008 (Measuring Computing, Norton, MA, USA) and TracedDaq software (Measuring Computing, Norton, MA, USA), by acquiring data in differential mode at +/− 4 V and 4 Hz.

### Whole cell protein extraction, nuclear/cytoplasmic fractionation and western blot analysis

Whole cell protein extraction was performed in lysis buffer (KH2PO4 0.1M

pH 7.5, NP-40 1%, added with Complete protease inhibitors cocktail (Roche Diagnostics, Basel, Switzerland) and 0.1mM b-glycerolphosphate for 20 min. and cleared by centrifugation at 14000 RCF for 20 min. Nucelar/cytoplamic fractionation was performed by cell pellets were lysis in Hypotonic buffer (10 mM Hepes pH 7.9, 1.5 mM MgCl2, 10 mM KCl) supplemented with protease inhibitors cocktail (Roche Diagnostics, Basel, Switzerland), and centrifuged to separate the cytoplasmic fraction from the nuclear pellet. Nuclei were lysed in a 1:1 mixture of Low Salt Buffer (20 mM Hepes pH 7.9, 25% glycerol, 0.2 mM EDTA, 20 mM KCl, 1.5 mM MgCl2) and High Salt Buffer (20 mM Hepes pH 7.9, 25% glycerol, 0.2 mM EDTA, 1.2 M KCl, 1.5 mM MgCl2), both supplemented with protease inhibitors cocktail (Roche Diagnostics, Basel, Switzerland), with nuclear extracts obtained by high speed centrifugation at 12,000 RPM at 4°C for 30 minutes.

Protein extract was quantified spectrophotometrically with the Bio-Rad

Protein Assay (Bio-Rad Laboratories, Hempstead, UK).

The same amount of proteins was separated in Laemmli loading Dye (2% SDS; 8% glycerol; 62,5 mM TRIS HCL PH 6,8; 0,005% bromophenol blue and 2% b-mercaptoethanol) by SDS PAGE in a polyacrilammide gel in Running Buffer ( 2,5 mM Tris, 19,2 mM Glycine and 0,1% SDS) at constant 30 mA for 1 h. Proteins were then transferred on a PVDF membrane (millipore) by a semydry transfer system with Transfer Buffer (2,5 mM Tris, 19,2 mM, 20% metOH) for 1,30 h. Employed antibodies are listed in supplemental materials and methods section.

### Co-immunoprecipitation assay

Immunoprecipitation assay was performed by cell lysis on ice in immunoprecipitation buffer (25 mM Tris HCl, pH 7.5, 150 mM KCl, 5 mM MgCl2, 1 mM EGTA,

10% glycerol, 0.8% Igepal/NP40, 0,4 U/μl RNAsi OUT and protease inhibitors cocktail from Roche Diagnostics, Basel, Switzerland). The lysates were cleared by centrifugation and quantified by Bradford protein assay (Bio-Rad Laboratories Hempstead, UK). Equivalent amounts (200 μg) of protein were incubated at 4°C with rotation overnight in immunoprecipitation buffer with 3 μg of anti-MDM2 (H-221; Santa Cruz Biotechnology, Santa Cruz, CA, USA) Protein A/G-coated agarose beads (Santa Cruz Biotechnology, Santa Cruz, CA, USA) were added to the extracts and mixed by rotation for an additional 3 hours at 4°C. The beads were washed four times with immunoprecipitation buffer. At the end of the final wash, beads were resuspended in Laemmli loading buffer for western blot analysis or in TRIreagent (Ambion, Austin, TX, USA) for RNA extraction. The extracted RNA was divided in two fractions and used directly for total 5S rRNA analysis or to retrive the 5-EU labeled and the unlabeled RNA fraction by using the nascent RNA capture kit^®^ (Life technologies, Eugene Oregon, USA), as indicated after.

### Neo-synthesized and pre-existing 5S rRNA evaluation

Neo-synthesized and pre-existing RNA amounts were evaluated by Nascent RNA Capture Kit^®^ (Life technologies, Eugene Oregon, USA) following manufacturer's instructions. In particular, HCT116 cells were seeded in 100 mm culture dishes and were labeled by 5-Ethynyl Uridine (5-EU) at 50 μM for the indicated times after 72 hours from the beginning of RNA interference. Cells were then collected after a pulse labeling of 1 hour and a chase time of 2 hours with 1 mM Uridine or after a continuous 5-EU labeling (8 hours) in presence or absence of 8 nM ACTD. At the end of the treatments, RNA was extracted with TRIreagent (Ambion, Austin, TX, USA) from whole cells (for global 5S RNA neosynthesis evaluation) or from MDM2 immunoprecipitated samples (for the measurement of neo-synthesized and pre-existing 5S rRNA bound to MDM2). The same amount of RNA (200 ng) was used directly for total fraction evaluation or was modified via click chemistry. Click chemistry based biotinylation was carried out in click-it reaction buffer (25 μl Click-iT EU buffer, 4 μl 25 mM CuSO4 and 1,25 μl of 10 mM Biotin azide, reaction buffer1, reaction buffer 2 and reaction buffer 3 only after 3 minutes) for 30 min at room temperature. Biotinylated RNA was precipitated by ammonium acetate for 10 minutes. After 10 minutes of centrifugation at 14000 RCF the pellet was washed twice with ETOH at 75%. All the retrieved RNA was then bound to 15 ul of streptavidin magnetic beads (Dynabeads, Life technologies, Eugene Oregon, USA) for 30 minutes at room temperature while gently shaking. The unlabeled RNA fraction (Pre-existing) was obtained by precipitation of the supernatant of streptavidin beads binding reaction. Beads was washed 5X 150 ul of Click it wash buffer 1 and 5 × 150 ul of Click it wash buffer 2, resuspended in 15 μl of wash buffer 2 and in bead reverse-transcribed for labeled RNA evaluation. The captured labeled RNA (neo-synthesized), the unlabeled RNA (pre-existing) and the total RNA were reverse-transcribed in cDNA by using High capacity reverse transcription kit (Life technologies, Eugene Oregon, USA and Real time qPCR was then performed for 5S rRNA evaluation. Fold Changes of neo-synthesized or pre-existing 5S rRNA populations were calculated in relation to the fold changes of the total 5S rRNA immunoprecipitated with MDM2. For each sample the following formula was applied: % Neo = ΔctNeo * (ΔctNeo + ΔctPre)/Δct TOT;% Pre = 2E−ΔCT Pre * (2E−ΔCT Neo + 2E−ΔCT Pre)/ 2E−ΔCT TOT. Δct TOT was relative to the maximum 5S rRNA amount measured (ACTD 8 h), while ΔctNeo and ΔctPre were relative to the background measured in the NS IGG immunoprecipitated sample.

Statistical analysis on the data obtained was performed by using the paired *T*-Test on three experimental replicates by considering significative data with a *P value* < 0,05.

## SUPPLEMENTARY MATERIALS FIGURES



## Abbreviations

5SRNP: Pre-ribosomal complex RPL5/RPL11/5S rRNA
ACTD: Actinomycin D
siRNA: Small interfering RNA
MDM2: Mouse Double minute 2
POLI, POLII, POLIII: RNA Polymerase I, II, III
5-EU: 5- Ethynyl Uridine
