# Supplementary material for: The pre-existing population of 5S rRNA effects p53 stabilization during ribosome biogenesis inhibition
Source: Oncotarget. 2016 Dec 9;8(3):4257–67. doi: 10.18632/oncotarget.13833 (PMC5354829; doi:10.18632/oncotarget.13833)
Supplement: Supplementary file 1 [file oncotarget-08-4257-s001.pdf]

The pre-existing population of 5S rRNA effects p53 stabilization during ribosome biogenesis inhibition

Supplementary Materials

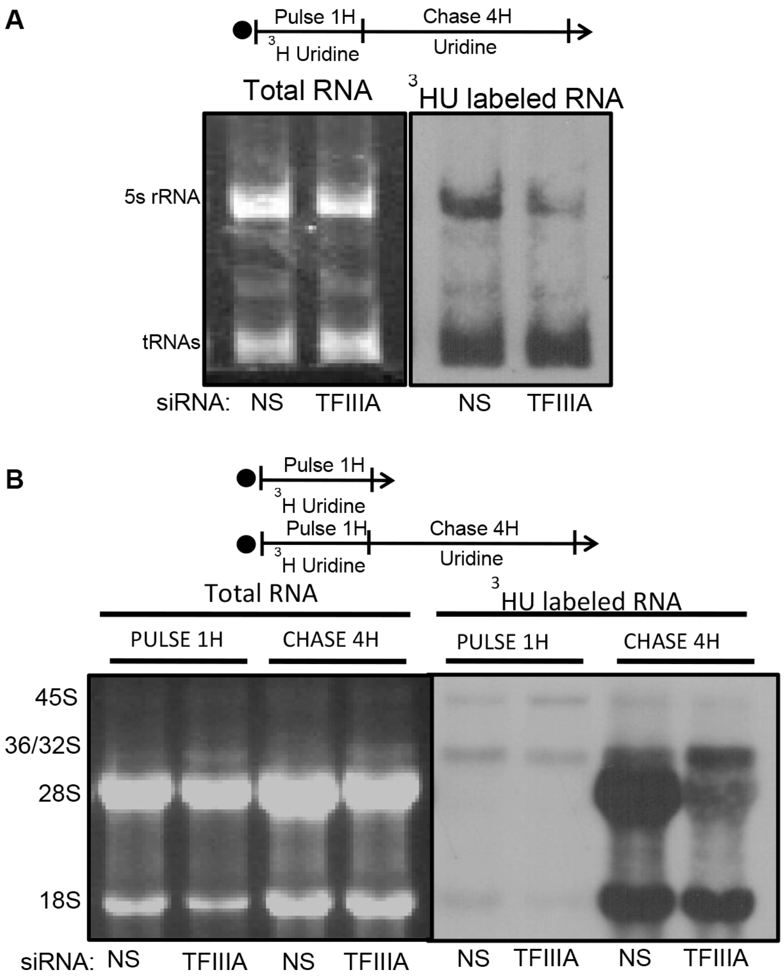

Supplementary Figure S1: Autoradiographic analysis of ^{3}H Uridine labeled HCT116 cells were treated with siTFIIIA or siNS, after 72 hours from the beginning of the transfection procedure. Cells were then pulse labeled for 1 hour and RNA was extracted after a chase time of 4 hours, as indicated above by the representation of the experimental design. Left panel: ethidium bromide stained total RNA; Right panel: autoradiographic exposition of ^{3}H Uridine labeled RNA. The RNA was extracted following the above indicated experimental design to analyze (**A**) Neosynthesis of 5S rRNA by 10% Acrilammide TBE/UREA elettrophoresis (**B**) Ribosomal RNA processing by 1% Agarose gel elettrophoresis

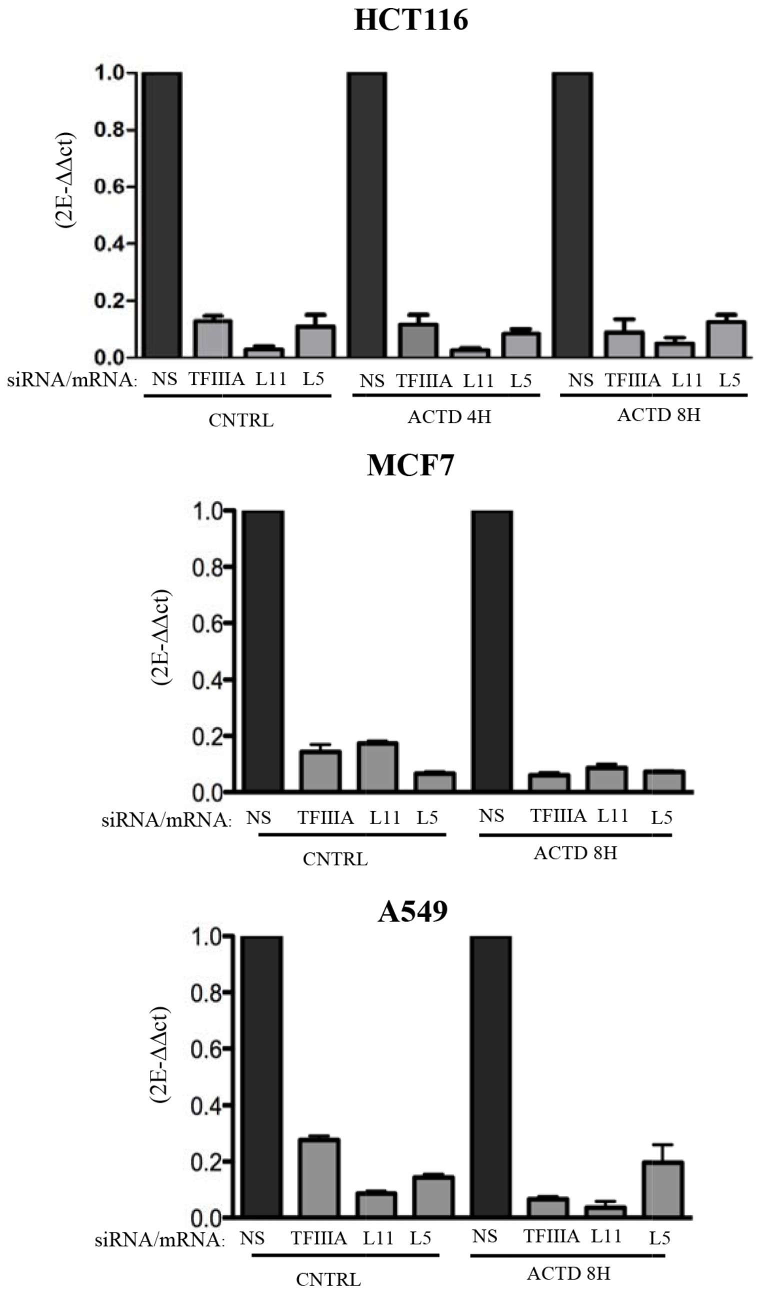

Supplementary Figure S2: Efficiency of TFIIIA RPL11 and RPL5 silencing. Real time qPCR analysis relative to the indicated mRNA target expression in HCT116, MCF7 and A549 cells, as indicated. Graph bars represents mean ± SEM of three independent experiments.

Alterantive siRNA sequences

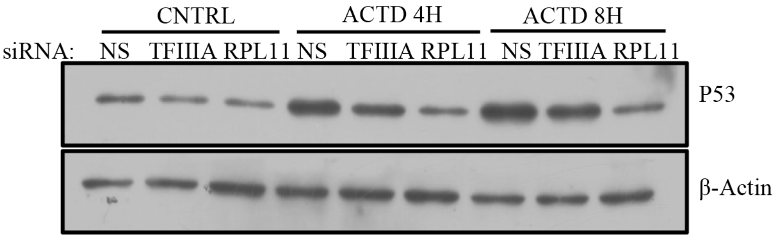

Supplementary Figure S3: Effect of TFIIIA and RPL11 silencing on p53 stabilization (alternative siRNA sequences). Western blot analysis of p53, and β-actin as loading control, in HCT116 cells, which were treated with ACTD for 4 or 8 hours at 8 nM,72 hours from the beginning of siTFIIIA and RPL11 or siNS transfection procedure. Select stealth RNAi (Thermo scientific) were used as alternative siRNAs. (siTFIIIA: cat.HSS104587; siRPL11 cat. HSS109330; siNS cat. 12935300).

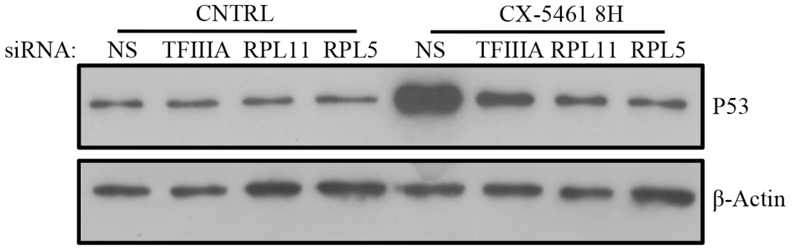

Supplementary Figure S4: Effect TFIIIA and RPL11 silencing on p53 stabilization. Western blot analysis of p53, and β-actin as loading control, in HCT116 cells, which were treated with the non-intercalating RNA Polymerase I inhibitor CX-5461 (Selleckchem, Huston, TX, USA) for 8 hours at 1 mM,72 hours from the beginning of siTFIIIA and RPL11 or siNS transfection procedure, as indicated.

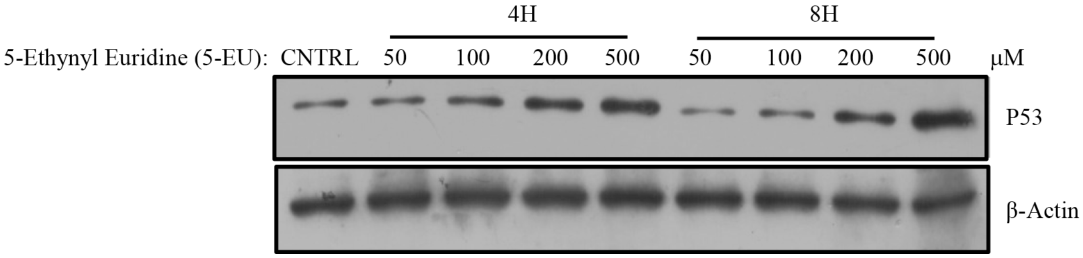

Supplementary Figure S5: Effect of 5-Ethynyl Euridine on p53 stabilization. Western blot analysis of p53, and β-actin as loading control, in HCT116 cells, which were treated with RNA labelling agent 5-Ethynyl Euridine (Thermo Scientific) for 4 or 8 hours hours at the indicated concentrations.

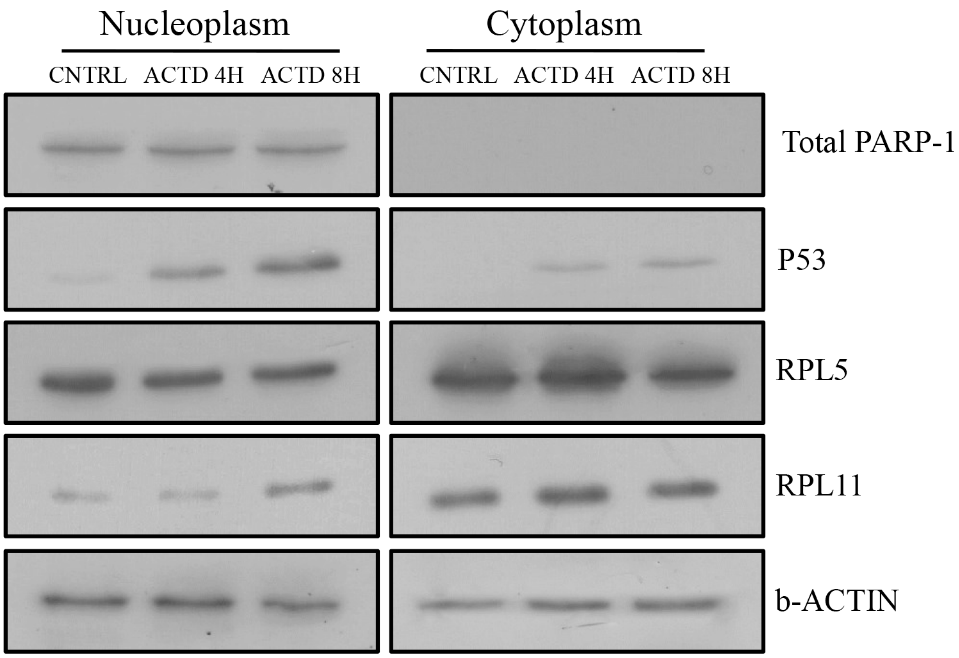

Supplementary Figure S6: Analysis of nuclear distribution of RPL5 and RPL11 during ribosome biogenesis inhibition. Western blot analysis of P53, RPL11 and RPL5 in nuclear or cytoplasmatic extracts of HCT116 cells after progressive inhibition of ribosome biogenesis by ACTD (8 nM) treatment for 4 or 8 hours. β-actin was used as general loading control, while PARP-1 as a nucleat marker.

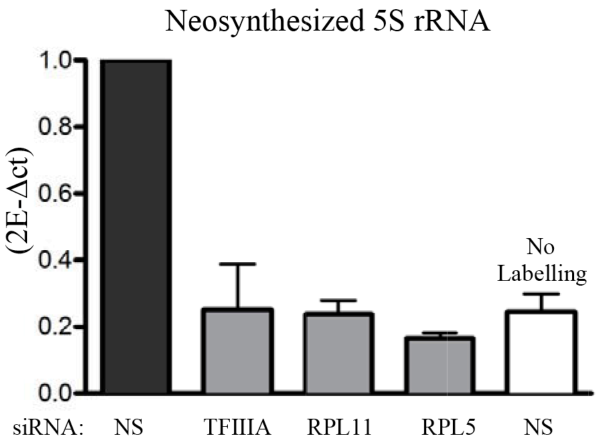

Supplementary Figure S7: Analysis of 5S rRNA neosynthesis after TFIIIA, RPL11 and RPL5 interference Real time qPCR of neosynthesized 5S rRNA expression. 72 hours after the beginning of TFIIIA, RPL11 and RPL5 interference HCT116 cells were labeled with 5-EU for 1 hours and chased with Uridine for 2 hours. The extracted RNA was modified with biotin azide, captured by streptavidin magnetic beads, reverse-trascribed into cDNA and 5S rRNA relative sequence was amplified by qPCR. Graph bars represents mean ± SEM of three independent experiments.

siRNA sequences: related to cell lines, culture conditions and siRNA transfection section

**TFIIIA**	5′-CACUAGGCAUGCUGUUGUA-3′
**RPL11**	5′-AAGGTGCGGGAGTATGAGTTA-3′
**RPL5**	5′-ACGCUUGGUGAUACAAGAUAA-3

Primer sequences: related to RNA extraction, reverse transcription and Real Time qPCR section

**TFIIIA**	**FOR:** 5′-TGAGATGAGAGGCCAAACTCCGTT-3′<br>**REV:** 5′-TTGTGTGTGAACATGCTGGCTGTG-3
**RPL11**	**FOR:** 5′-TCCACTGCACAGTTCGAGGG-3′<br>**REV:** 5′- AAACCTGGCCTACCCAGCAC-3′
**RPL5**	**FOR:** 5′-GGTGTGAAGGTTGGCCTGAC-3′<br>**REV:** 5′-GGCACCTGGCTGACCATCAA-3′
**5S rRNA**	**FOR:** 5′-GGCCATACCACCCTGAACGC-3′<br>**REV:** 5′-CAGCACCCGGTATTCCCAGG-3′

Antibodies: related to whole cell protein extraction, nuclear/cytoplasmic fractionation and western blot analysis section

**α-P53**	Clone BP53-12, Novocastra Laboratories, Newcastle Upon Tyne, UK
**α-RPL11**	Clone 3A4A7, 37-3000, Thermo Fisher/Life Technologies, Waltham, MA USA
**α-RPL5**	Polyclonal, A303-933A, Bethyl Laboratories inc., Montgomery, TX, USA
**α-PARP-1**	Clone 9542, Cell Signaling Technology, Beverly, MA, USA
**α-βActin**	Clone AC-74, A2228, Sigma-Aldrich,Saint Louis, MO, USA
**α-MDM2**	Clone SMP14 sc-965 (WB), clone H-221(IP), sc-7918, Santa Cruz Biotechnology, Santa Cruz, CA, USA
